# Supplementary material for: Constraints on the G1/S transition pathway may favor selection of multicellularity as a passenger phenotype
Source: eLife. 2026 Jun 18;15:RP109833. doi: 10.7554/eLife.109833 (PMC13278735; doi:10.7554/eLife.109833)
Supplement: Peer review history

# Editor report: eLife Assessment

**DOI:** 10.7554/eLife.109833.3.sa0

**Authors:** Vincent J Lynch

**Affiliations:** University at Buffalo, State University of New York United States

**Keywords:** Convincing, Important

This **important** study implicates that changes in cell regulation may contribute to the evolution of multicellularity. The evidence supporting the conclusions is **convincing**, with rigorous methods used to test alternative hypotheses. The work will be of broad interest to cell and evolutionary biologists and those studying the cell cycle and cancer.

# Referee report: Reviewer #1 (Public review)

**DOI:** 10.7554/eLife.109833.3.sa1

Summary:

Ducrocq et al. present research exploring the genetic link between simple multicellular group formation (ace2Δ/ace2Δ) and its interaction with cell-cycle progression mutants (e.g., cln3Δ/cln3Δ), demonstrating that this combination can provide fitness benefits during fluctuating resource conditions, resulting in a rapid increase in the fraction of multicellular cell-cycle mutants over unicellular yeast without selection for multicellular size. Because both the multicellular phenotype and the regulatory link enabling faster escape from the stationary phase are controlled by the ACE2 transcription factor, this work demonstrates that multicellular cluster formation can arise as a side effect of a completely independent fitness advantage unrelated to the benefits of group formation itself. As a "passenger phenotype," multicellularity could thus emerge for other selective reasons, potentially facilitating a later transition to more entrenched multicellularity if novel conditions arise that make multicellular group formation directly beneficial.

Importantly, while the literature generally assumes that multicellular group formation incurs a cell-level fitness cost, this work demonstrates that certain genetic - environmental interactions can confer fitness benefits even at the level of individual cells forming multicellular groups. This finding should inspire both theoretical and empirical work exploring multicellular group formation selected for benefits at the level of individual cells, rather than the benefits of forming a larger organismal size that most work has relied on so far.

Strengths:

This work is novel and exciting for research exploring the very first steps of the transition from unicellularity to simple multicellularity. The formation of multicellular groups is almost always assumed to come at a cell-level fitness cost due to reduced reproductive fitness compared to remaining unicellular, which generally needs to be outweighed by the benefits of multicellular group formation (e.g., large size to escape predation) for the multicellular phenotype to be stable. However, this study presents an interesting case of a genetic and environmental condition under which individual cells forming simple multicellular clusters can actually have higher reproductive fitness than solitary living yeast cells. This contrasts with previous snowflake yeast studies where the multicellular phenotype was primarily beneficial due to strong selection for large groups (rather than cell-level fitness gains).

The claims and interpretation of the results align well with the data presented. This is due to the careful and straightforward experimental design testing predictions with a clear, stepwise methodology. The authors rule out alternative explanations and provide support for the proposed link between the mutations (ace2, cln3, and others), their impact on faster exit from quiescence and earlier entry into reproduction in fresh media, and the resulting higher fitness in the snowflake yeast phenotype compared to unicellular yeast.

This experimental framework (combining cell-cycle mutants under the same multicellular background) is very much likely to be adopted by others in the community to explore downstream implications of these results in laboratory and environmental yeast isolates.

Weaknesses:

The authors show that the same multicellular phenotype with higher cell-level fitness due to faster exit from the stationary phase can also be observed with alleles found at other loci in non-laboratory yeast strains, implying that the results are likely not specific to a peculiar case genetically engineered in laboratory strains, but that similar phenotypes may be present in nature. However, this remains to be explored by examining the natural ecology of commercially available or wild yeast isolates and their genomes. This is not a weakness of this study per se, but rather a direction for future work. It does mean, however, that the relevance of these findings for early multicellularity in yeast, and even more so for nascent multicellularity in distinct taxa, remains to be explored in the future. Until then, it is difficult to make strong claims about how applicable these results would be for non-laboratory yeast and other taxa. Regardless, this work represents a very exciting finding.

Comments on revised version:

The authors addressed all concerns thoroughly.

# Author comment: Author response

**DOI:** 10.7554/eLife.109833.3.sa2

**Authors:** Tom Louis Ducrocq, Damien Laporte, Bertrand Daignan-Fornier

**Affiliations:** Centre National de la Recherche Scientifique Bordeaux France; Institut de Biochimie et Génétique Cellulaires, CNRS Bordeaux France; Centre National de la Recherche Scientifique Bordeaux France

The following is the authors’ response to the original reviews.

**Public Reviews:**

**Reviewer #1 (Public review):**

Summary:

Ducrocq et al. present research exploring the genetic link between simple multicellular group formation (ace2Δ/ace2Δ) and its interaction with cell-cycle progression mutants (e.g., cln3Δ/cln3Δ), demonstrating that this combination can provide fitness benefits during fluctuating resource conditions, resulting in a rapid increase in the fraction of multicellular cell-cycle mutants over unicellular yeast without selection for multicellular size. Because both the multicellular phenotype and the regulatory link enabling faster escape from the stationary phase are controlled by the Ace2 transcription factor, this work demonstrates that multicellularity can arise as a side-effect of a completely independent fitness advantage unrelated to the benefits of group formation itself. As a "passenger phenotype," multicellularity could thus emerge for other selective reasons, potentially facilitating a later transition to more entrenched multicellularity if novel conditions arise where group formation becomes directly beneficial.

Strengths:

This work is novel and exciting for research exploring the very first steps of the transition from unicellularity to simple multicellularity. This is particularly significant because the formation of multicellular groups is almost always assumed to come at a cell-level fitness cost due to reduced reproductive fitness compared to remaining unicellular. This cell-level fitness cost generally needs to be outweighed by the benefits of multicellular group formation (e.g., large size escaping predation) for the multicellular phenotype to be stable, which is true for a large number of cases studied in the literature, where the multicellular phenotype can only evolve over unicellular competitors under strong selection for multicellular groups. However, this study presents an interesting case of a genetic and environmental condition under which individual cells (forming simple multicellular clusters) can actually have higher reproductive fitness than unicellular yeast. This demonstrates that the assumed cost at the single-cell level does not always apply. In summary, this work represents a unique example contrary to common assumptions regarding the costs of multicellular phenotypes, showing that simple multicellular phenotypes can evolve and remain stable without requiring strong selection for multicellular size or other benefits of group formation.

The claims and interpretation of the results align well with the data presented. This is due to the careful and straightforward experimental design testing predictions with a clear, stepwise methodology, ruling out alternative explanations and providing support for the proposed link between the mutations (ace2, cln3, and others), their impact on faster exit from quiescence, and thus earlier entry into reproduction in fresh media, resulting in higher fitness in the snowflake yeast phenotype compared to unicellular yeast.

Weaknesses:

The authors show that the same multicellular phenotype with higher cell-level fitness due to faster exit from the stationary phase can also be observed with alleles found at other loci in non-laboratory yeast strains, implying that the results are likely not specific to a peculiar case genetically engineered in laboratory strains, but that similar phenotypes may be present in nature. However, this remains to be explored further by examining the natural ecology of commercially available or wild yeast isolates and their genomes. This is by no means a weakness of this study and, therefore, not necessarily something the current work can improve. It does mean, however, that the relevance of these findings for early multicellularity in yeast, and even more so for nascent multicellularity in distinct taxa, remains to be explored in the future. Until then, it is difficult to make strong claims about how applicable these results would be for non-laboratory yeast and other taxa. Regardless, this work does its part by representing a very exciting finding.

**Reviewer #2 (Public review):**

Summary:

Here, the authors attempt to demonstrate that a simple model of multicellularity - snowflake yeast - exhibits key ecologically relevant changes in the regulation of the cell cycle. By examining the effects of the ace2 mutation in environments where multicellularity is not directly selected for or against, and combining it with mutations in key cell cycle regulators, they hope to show that mutations driving simple multicellularity can be selectively favored due to their effects on the release from quiescence rather than their effects on multicellularity itself.

Strengths:

The experiments performed are extensive and thorough. The yeast genotypes examined are judiciously chosen, so as to map out a functional model of the relationship between alterations to cell cycle control and changes to multicellularity phenotypes. Multiple possible interactions are examined, with the causal link and model of the relationship between the multicellular passenger phenotype and the selectable quiescence-release phenotype being well-supported. There are extensive controls demonstrating the separation between the 'passenger' multicellular phenotype and the cell cycle regulation phenotypes examined, including haploid/diploid strains with different multicellular phenotypes but similar cell cycle regulation phenotypes, and phenocopy strains in which downstream enzymes are deleted rather than key central regulators.

Weaknesses:

My only concerns about these results relate to the focus on selection on cell cycle control being examined in a model of multicellularity with key core cell cycle mutations rather than in a wild-type background, as this is a somewhat artificial system.

I believe, however, that the authors convincingly make their case that this work on the multicellular phenotypes of yeast represents a potent proof-of-concept that simple multicellularity can be driven into existence or selected for as a passenger phenotype due to pleiotropic effects of mutations under selection from real-world ecological pressures. They are able to connect this phenotype back to known mutations of particular cell cycle regulators (RB) in other multicellular lineages and demonstrate that ecologically relevant changes to the cell cycle are connected to multicellular phenotypes. As a proof of concept of the connection between these phenotypes, rather than a study of a particular event in the past of a living lineage, it makes a strong case.

A longstanding question in the field of multicellularity is the selective pressures that can drive simple multicellularity into existence and then act on simple multicells to drive their increased size and complexity. This work brings to the table tangible evidence of the possibility that, instead of being selected for on its own, simple multicellularity can be a side-effect of selection on other key phenotypes.

This separates the question of the origins of multicellularity and the forces that drive its further evolution. This separation can reframe how the field is studied, especially in the context of the apparent dichotomy between dozens of origins of 'simple' multicellularity across the tree of life and a few origins of 'complex' multicellularity in the history of Earth. Especially in light of other evidence that multicellularity is connected to changes in cell cycle regulation, I believe that this is an important insight that will alter the way we think about the origins of this key evolutionary transition.

We thank the reviewers for their insightful comments on our work.

We agree with reviewer #1 that further experiments would be needed to figure out how the observations done on lab strains can apply to yeast in various ecological conditions and particularly in the wild. We here provide a proof of principle that multicellularity selection can arise as a side-effect. It obviously does not prove that it took place during yeast evolution, but we would like to emphasize that resource fluctuations are very common in ecological conditions, making it highly likely that the environmental conditions necessary for the selection of the side effects described have arisen.

We agree with reviewer #2 that our work on yeast strains is “somewhat artificial” as often the case with model organisms under laboratory conditions. Importantly though, we showed that the effect found with the *cln3* knock-out mutation can be phenocopied by overexpression of *WHI5* (encoding the yeast equivalent of Rb). We propose that variations in the levels of cell cycle regulators during evolution may have played a role in multicellularity selection as a side effect. We agree that this is merely a hypothesis to explain the selection of multicellularity (just like predator escape) and that there is no direct evidence that this occurred in the history of the lineage. Nevertheless, our work provides a first evidence that such a selection of multicellularity as a side effect could be possible, and gives a framework to understand how multicellularity can persist in the wild, even when it is not the primary target of selection.

**Recommendations for the authors:**

**Reviewer #1 (Recommendations for the authors):**

As mentioned in my public review, I very much appreciate this work, its interpretation for early multicellularity as an example opposite to the assumed cost of multicellular phenotypes, and the robust design behind the premise and claims. Therefore, my suggestions below are mostly aimed at improving the readability and data presentation.

(1) In the abstract, Lines 24-27 (the last sentence): This statement is worded too generally and therefore reads as too strong. I think the authors' work provides an example that multicellularity itself does not need to be beneficial all the time - this is really exciting and makes sense! However, there is a substantial body of work showing the origin and maintenance of multicellularity for its direct benefits. Relative to that body of work, this represents a special case, and therefore, while we should definitely reconsider the view that "multicellularity always comes at a cell-level fitness cost," we cannot overgeneralize these findings. Please consider reframing this statement.

Done, now line 25 (addition of “in some cases”)

(2) Line 48 (Introduction): "This mostly concerns two major regulators, RB and Cyclin D." Which organisms are you referring to? Please specify.

Done.

(3) In the Introduction, there are at least three sentences that need citations: L57-58, L59-60, and L65. For instance, I do not know what makes CLN3 the yeast functional equivalent of RB, and I wanted to verify this claim, but no references are cited. Please ensure citations are provided throughout the manuscript.

Done: ref 11,12 and 13 were added

(4) This is my main request regarding data collection and presentation. The authors share some microscopy images of mutant strains in Figure 2 for different purposes (e.g., Figure 2B compares the fraction of budded cells between two genotypes). However, I would appreciate seeing a collected microscopy figure showcasing the phenotypes of all genotypes that went into competition experiments, including the planktonic (WT lab strain) yeast, either where they appear or in a supplementary figure, all presented with the same magnification and scale to make them comparable. Because cell size, shape, and multicellular phenotype are all key aspects of the competition experiments, being able to see all those genotypes/phenotypes would prepare the reader to make predictions about the fitness assays and other experiments.

Done Supplementary Figure 1 B-E were added

(5) Related to my previous point, I would appreciate seeing cell size measurements for the different genotypes (both single cells of planktonic genotypes and single cells forming multicellular clusters). Cell size is a key trait that directly impacts the results shown in the paper, and summary statistics comparing them would be helpful for interpreting the results.

Done Supplementary Figure 1 F was added

(6) In competition experiments, the authors mix unicellular and multicellular yeast clusters at 50/50 and measure the fraction of a phenotype of interest (usually the % of snowflake). It took me a while to understand what is being counted under the "% snowflake yeast" category. This is because, while each cell in unicellular yeast should be counted as one unit, one can count a snowflake yeast composed of 50 cells as 50 units or as 1 unit. Please clearly state what is being counted for the Y-axis labeled "% of snowflake yeast" (or relabel those Y-axes in plots to make this clear).

Done: Added in figure legend 1A and Y-axes of competition figures

(7) I recommend editing the genotype labels in figures (see, for instance, Figure 1B, C, D). In Figure 1B, the bars are labeled as "CLN3/CLN3 co-culture" or "cln3Δ/cln3Δ co-culture," etc. These are actually co-cultures of SF vs. PK (with or without a CLN3 copy). Please consider using more representative labels that will be easier for readers to understand.

Done: this has been changed in all concerned figures

(8) In the Results, L225, you begin referring to AMN1368D as AMN1. I suggest using the full allelic form throughout the text so it will be clear each time that you are referring to that specific allele, as I was confused about whether you were discussing the allele or the gene AMN1 itself.

This has been changed throughout the text.

(9) Discussion, Lines 250-252, states that this is a "situation that is likely to happen very often under ecological conditions." Are there any examples you can cite?

Done, as also requested by reviewer #2 (now line 256-7)

(10) Lines 272-275 contain a strong, general statement suggesting that co-evolution of cell cycle regulation and multicellularity could be more general (which is acceptable as speculation). However, the suggestion that this co-evolution could have "started very early in the evolution of eukaryotic cells" is too speculative. I would recommend sticking with the alternative, suggesting that the link between the two phenotypes may be a case of convergent evolution.

Done

(11) Lines 278-279 are both vague and too bold. The text mentions a link between cancer and multicellularity and then extends this link through cell cycle regulators. Without explaining the connection between cancer and multicellularity and then trying to link it to cell cycle regulators, all in a few words without background, this sentence is too vague. Please consider deleting this or spending more time clearly explaining the link, which would at best still be speculative.

These speculative sentences were removed.

(12) First, I wanted to note that I highlighted Lines 284-287, as this passage is clearly written and provides a nice argument. I also wonder if you could mention that your work shows simple multicellular cluster formation should not always come at a cost, contrary to the general assumption in the literature, and add a few citations to support that claim. This would highlight how significant this work is within the broader multicellularity literature.

Changed in discussion (now line 242-4 with additional references 30 and 31)

(13) I recommend labeling the genotype of your "quintuple mutant" in Figure 3. You can refer to it as the quintuple mutant in the text, but I had to go back and forth to see what those mutations were when trying to think about potential genetic interactions. Even the legend of Figure 3 does not specify the genotype and refers to it only as the "quintuple mutant."

Now explicitly stated in the title of the figure

**Reviewer #2 (Recommendations for the authors):**

I find the presented research to be of high quality, with very important implications. I have suggestions for improvement of the manuscript, but they are largely stylistic, with one paper that I believe deserves citation regarding the proteins involved. I see little need for additional experiments or analysis, just a clearer description of the results and their significance.

(1) Line 62: Yeast CLN3 definitely performs the same role as cyclin D in the cell cycle, but has an unclear phylogenetic relationship with the rest of the cyclins. See Cross, Buchler, & Skotheim 2011 ("Evolution of networks and sequences in eukaryotic cell cycle control"). This reference also covers the functional relationship between RB and Whi5, referred to in nearby sentences, as does Medina, Walsh, and Buchler 2019 ("Evolutionary innovation, fungal cell biology, and the lateral gene transfer of a viral KilA-N domain").

The reference has been added

(2) Line 69: Is the question whether the evolution of G1/S regulation favoring multicellularity the question, or the two of them being connected such that the evolution of one can affect the other?

It is clearly the first of the two questions.

(3) Line 73: Comma after Ace2.

Done

(4) Line 76: It would be clearer to specify that snowflake and ACE2 yeast were co-cultured without settling selection or other selection that explicitly favors multicellularity, unlike in experiments where multicellular evolution is observed, as in Ratcliff publications.

This is now specified.

(5) Line 80: Specify which phenotypes observed for ace2 mutants are observed, specifically, both the multicellularity and the release from quiescence.

Done

(6) Line 146: This observation should be noted as another indication that the multicellular phenotype is not behind the selective pressure, because it is so different between unicells and multicells.

Overall, you have very strong evidence that this is the case, and emphasizing this would benefit the paper!

Done.

(7) Line 151: specify that you are maintaining yeast in proliferation in coculture.

Done.

(8) Line 181: This is another key experiment showing that the multicellular phenotype is not the causal reason for the change in quiescence. It might make things clearer to bring all these confirmatory experiments together, particularly the haploids and the sonicated single cells.

This is now clearly stated line 195.

(9) Line 225: The choice of referring to the non-laboratory strain as the 'AMN1' wild type default may be confusing to readers, who may treat the genetic background you are using as the ground truth wild type. I recommend throughout the paper always specifying the allele's amino acid to avoid any confusion.

The genotype is now clearly presented throughout the text.

(10) Line 238: I would continue to specify that the multicellular phenotype has no selective advantage, specifically when no selection for size is applied.

See added sentence Line 242-4 (revised version)

(11) Line 243: I would say that the evolution of cell cycle regulation may interact with the multicellular phenotype.

This was changed (now line 248)

(12) Line 244: Strike 'indeed' and the 'the' before AMN1 and ACE2.

Done

(13) Line 252: Suggest some ecological conditions under which quiescence exit is likely, such as boom and bust or moving from rotting fruit to rotting fruit.

Done

(14) Line 267: Are you suggesting that the specific genes AMN1 and ACE2 had particular effects on actual organisms in the past, or that it represents a broad pattern of evolution in which multicellularity could be more broadly related to exit from quiescence? I believe it is the latter, and I think that should be clearer.

Modified as suggested

(15) Line 280: In this paragraph, I think that the point being made could be slightly clearer - if I am not mistaken, you are making the distinction between the appearance of multicellularity and its refinement under selection, and that the former may be more common than previously believed, given this proof of concept. I think this can be made clearer. Furthermore, it is worth noting that all experiments that show effects of the multicellular phenotype are in mutant backgrounds, and explaining why this is still relevant to wild organisms. It might be taken by some as indicating that the multicellular phenotypes are not relevant to a wild population, but the connection to known RB mutations in known multicellular lineages and the fact that it is connected to a very key aspect of cell cycle regulation, I think, overcomes this issue, and this should be made clear.

Our study reveals a genetic link between multicellularity and Whi5 and Cln3, two important G1/S cell cycle regulators. Similar genetic interactions have been observed in phylogenetically distant species, reinforcing the idea that the interplay between cell cycle regulation and multicellularity is a general feature and not a mere artifact of mutant background.

The neutral fitness effect of multicellularity in wild-type backgrounds is particularly of interest. By being maintained as a side effect of selection on fundamental cellular processes, the neutral effect of multicellularity may have provided “an evolutionary scheme” for its repeated emergence throughout the tree of life. As such, the "passenger selection" hypothesis fits well with the observations of phenotypic reversibility and facultative multicellularity, despite varying and specific selective pressures. Our work thus gives a framework to understand how multicellularity can persist in the wild, even when it is not the primary target of selection.

(16) Line 314: What promoters are they driven by?

Specified

(17) Line 336: What was the culture volume, and the volume transferred?

Specified

(18) Line 362: How was the proportion of blue-stained cells scored? Manually, or with an imaging software cutoff?

Specified

(19) Figure 1: I think that the full genotypes of each strain should be specified, either in the legend or the key of the figure, rather than always specifying the ACE2 genotype and other mutations separately.

Done as requested by reviewer #1

(20) Figure 2E, 2F: Same as Figure 1, regarding genotypes.

Done
